# The Effect of Abusive Supervision on Employee Creativity: The Mediating Role of Negative Affect and Moderating Role of Interpersonal Harmony

**DOI:** 10.3389/fpsyg.2022.796355

**Published:** 2022-03-10

**Authors:** Lili Chen, Zhixiao Ye, Zahid Shafait, Hongying Zhu

**Affiliations:** ^1^Property Management Department, School of Management and Institute of Modern Services, Zhejiang Shuren University, Hangzhou, China; ^2^School of Management, Northwestern Polytechnical University, Xi’an, China; ^3^Institute of Modern Services, Zhejiang Shuren University, Hangzhou, China

**Keywords:** abusive supervision, employee creativity, negative emotions, interpersonal harmony, China

## Abstract

This study investigates the relationship between abusive supervision and employee creativity by shedding light on the mediating role of negative affect and the moderating role of interpersonal harmony. Based on affective events theory, it was hypothesized that abusive supervision impacts employees’ negative affect and their creativity. Data from a questionnaire survey of 398 Chinese employee–supervisor dyads were collected and analyzed. The results support our hypotheses, address unexplored theoretical predictions, and suggest that organizations should deal with the factors undermining employees’ emotions to improve their creativity.

## Introduction

In today’s volatile and complex business environment, one of the crucial challenges for organizations is how to enhance the creative abilities of their workers (Hirst et al., 2009). Employee creativity is defined as the propensity to generate new, useful, and novel ideas regarding products, practices, services, or procedures in the workplace (Jahanzeb et al., 2020). Considerable evidence has suggested that employee creativity can benefit organizational reformation and innovation, competitiveness, effectiveness, and survival (Nystrom, 1990; Zhou, 2003; Shalley and Gilson, 2004; Sijbom et al., 2018), and enhance long-term business success (Hon, 2012). Given the importance of creativity, how to cultivate and maintain employee creativity in organizations has been a hotspot for research on organizational behavior (Shafait et al., 2021a,b).

Literature has focused on identifying ways to promote employee creativity (Zhang et al., 2017; Liu et al., 2020). Leadership, an important component of the organizational environment, has been deemed an important antecedent of employee creativity (Zhou and Hoever, 2014). While research has demonstrated that positive leadership styles, such as empowering leadership (Zhang et al., 2018), transformational leadership (Dong et al., 2017), and servant leadership (Zhu and Zhang, 2019), can boost employee creativity, research has also shown that destructive leadership styles, such as abusive supervision, can impede it. Ahmad et al. (2020) found that negative leadership styles had an even longer-lasting and stronger impact on employees, compared with positive styles. Abusive supervision, as a destructive style of leadership, was classically defined by Tepper as the subordinate’s perceptions of “engaging in the sustained display of hostile verbal and non-verbal behaviors, excluding physical contact” (Tepper, 2000, p. 178).

Previous study on the effect of leadership on creativity has focused on cognitive mechanisms, such as breach of psychological contract (Parzefall and Salin, 2010), psychological distress (Tepper, 2000), psychological safety (Liu and Wang, 2020), psychology empowerment (Xia and Ji, 2017), creative self-efficacy (Rabbani and Sarmad, 2019), self-efficacy (Chen and Wang, 2017), and creative role identity (Yang et al., 2020). However, the influence of abusive supervision on employee creativity has not been fully explored (Akram et al., 2021). Han et al. (2017) showed that abusive supervision had an indirect negative relationship with employee creativity via its impact on emotional exhaustion. Emotions play a vital part in success and failure of personnel in routine assignments (Shafait et al., 2021c,d). Akram et al. (2019) found that abused employees usually suffer from emotional exhaustion and tend to promote counterproductive work behaviors. However, negative emotions/affect differ from emotional exhaustion. While emotional exhaustion refers to the feeling of energy depletion that results from extreme psychological demands (Han et al., 2017), negative emotions/affect refer to an unstable psychological state associated with sadness, anger, anxiety, pain, and fear, related to specific situations (George and Zhou, 2007). Previous studies have neglected these potential effects triggered by abusive supervision. There is clearly a gap in research regarding negative affect as a mediator in the link between abusive supervision and employee creativity. Several studies have indicated that employees’ negative affect might influence their perception of abusive supervision (Yagil et al., 2011). In this study, we adopt affective events theory (AET) to explain the direct effect of abusive supervision on employee creativity and the indirect effect of negative affect as a mediator.

The core of AET is that employees’ emotions are directly affected by work events. Emotional responses influence individual behaviors in two different ways. One is that emotional reactions directly influence employees’ behaviors; these are affect-driven behaviors. The other is that emotional reactions indirectly influence employees’ behaviors through influencing employees’ work attitudes, such as job satisfaction and organizational commitment; these are judgment-driven behaviors. This study uses AET to explain the role of abusive supervision in triggering employees’ negative affect at work. We propose that employees will feel negative emotional reactions that subsequently affect individual creativity as a consequence of abusive supervision.

We also draw on the literature on harmony to propose interpersonal harmony as a boundary condition in the process by which abusive supervision impacts employee creativity. China is characterized by a higher power distance and a traditional oriental culture. Harmony, equal to Chinese “*he*,” is an important characteristic that promotes and preserves harmonious relations in Chinese traditional culture. Farh et al. (1997) thought of interpersonal harmony as a discretionary behavior of employees, which avoids the pursuit of personal power and interests that may adversely affect others or the organization. A review demonstrated that culture impacts employees’ responses to various facets of their work environment in a cross-cultural context (Gelfand et al., 2007). Lawler et al. (2008) argue that culture probably plays a significant part in how subordinates react to the employee–supervisor link. According to these studies, it is likely that harmony plays a significant role in how individuals cope with abusive supervision in the workplace. This study extends research on interpersonal harmony by exploring how it may moderate the effect of abusive supervision.

Based on AET, this study explains how negative work events (abusive supervision) trigger individual emotional responses (employees’ negative affect), which then affects individual behaviors (employee creativity). Hence, this study applies AET to achieve three main objectives: to investigate the relationship between abusive supervision and employee creativity, to examine whether negative affect mediates the relationship between abusive supervision and employee creativity, and to explore whether interpersonal harmony buffers the impact of abusive supervision on employee creativity via negative affect. These relationships form our theoretical model: negative affect represents the potential mediator and interpersonal harmony represents the moderator of the relationship between abusive supervision and creativity.

This study contributes to the literature on abusive supervision, AET, employee creativity, negative affect, and interpersonal harmony in several ways. First, this study incorporates both the moderating and mediating mechanisms into a mediated moderation model, explaining both how and when abusive supervision undermines creativity, how negative affect mediates abusive supervision’s effects on employee creativity, and how interpersonal harmony moderates abusive supervision’s effects on negative affect. Second, this study attempts to integrate AET with a theoretical emotional model that accounts for how abusive supervision triggers employees’ negative affect and how employees’ negative affect hinders their potential creativity in the workplace. Third, this study extends the context of previous research on abusive supervision because China is a country with a rigid hierarchical system, where many supervisors habitually abuse their employees with the organization’s best interests in mind, which is a different context compared to previous studies (Chen et al., 2021).

## Theoretical Background

### Abusive Supervision and Employee Creativity

Mumford (2003) argued that creativity needs to be explained in terms of novelty and usefulness. Novelty refers to originality that is the production of something new. Usefulness refers to appropriate utilization of produced idea to resolve on hand issue. The past decade has witnessed growing research on the issue of abusive supervision in organizations. The harmful effects of perceived abusive supervision on employees’ behaviors have been diffusely documented. For example, studies have indicated a positive relationship between abusive supervision and unfavorable work attitudes, deviant behaviors (Aryee et al., 2007; Mitchell and Ambrose, 2007; Tepper et al., 2008; Thau et al., 2009), and organizational silence (Kang and Kwon, 2018). Abusive supervision has been shown to increase employees’ psychological pressure, such as tension and emotional exhaustion (Duffy et al., 2002), to decrease self-efficacy (Harvey et al., 2007) and to negatively impact job behaviors and attitudes (Duffy et al., 2002; Tepper et al., 2008). It is somewhat surprising that the number of American employees affected by abusive supervision is increasing. Tepper (2007) speculated that 13.6% of American workers might have experienced abusive supervision. Another study reported that 33.5% of employees experienced abuse by their leaders “quite often” and even “very often” (Aasland et al., 2010).

Creativity, concerning the generation of novel and potentially valuable ideas or thoughts about products, practices, services, procedures, or administrative processes, is widely considered the significant prerequisite of organizational innovation (Hon, 2012). A creativity componential model proposed that threatening critical assessments indicating incompetence are an important contextual element that undermines employee creativity in the workplace (Amabile et al., 1996). Abusive supervision is deemed as one kind of such critical assessment because it indicates in the form of an abusive manner that the supervisor is not satisfied with the employee’s performance (Tepper et al., 2011; Wu et al., 2020). Empirical studies have examined the relationship between abusive supervision and employee creativity. Zhang et al. (2014) showed that the destructive effects of external contexts, including abusive supervision, are important antecedent variables of employee creativity. Some studies have confirmed that abusive supervision directly reduces employee creativity (Liu et al., 2010, 2012). Others have verified that abusive supervision has indirect negative effects on employee creativity through the mediating role of intrinsic motivation and emotional exhaustion (Zhang et al., 2014; Han et al., 2017). However, inconsistent research results provide a new theoretical gap in the complex relationship between abusive management and employee creativity and performance (Jian et al., 2012).

### Negative Affect as a Mediator

Affective events theory was proposed by Weiss and Cropanzano (1996) and is widely used to understand the relationships among affective events, affective reactions and attitudes, and the behaviors of employers and employees in the workplace. AET aims at exploring the relationship between affective events experienced by members of an organization in the workplace and their attitudes and behaviors. AET, focusing on the structures, incentives, and consequences of individual emotional responses in the workplace, suggests that features of a work environment can lead to positive or negative work events and that the experience of these work events triggers an individual’s emotional response, which further affects individual attitudes and behaviors. AET explores the relationship between affective events in the workplace, affective reactions and attitudes, and behaviors experienced by an organization’s members. According to AET, employees’ emotional reactions at work follow the paradigm of events–emotion–attitude–behavior. The working environment leads to the occurrence of work events and work events are the direct cause of employees’ emotions (Weiss and Cropanzano, 1996). Based on AET, workplace characteristics such as leadership style can trigger positive or negative affective events that affect employees’ emotions. Scholars have conducted a large number of empirical studies on this issue. Rodell and Judge (2009) found that sources of employee pressure would motivate emotions, such as anger and anxiety, which could cause antiproductive behavior. Abusive supervision, as an unpleasant experience or perception in the workplace, is one such work event that can trigger emotional responses such as anger, frustration, distress, psychological depression, and other negative affect. Based on the above analysis, abusive supervision and employees’ negative affect are positively correlated.

The relationship between negative affects and employee creativity has been widely discussed. Amabile et al. (2005) showed that negative work events would cause employees to have negative affects and weaken their creativity. AET establishes the first relationship between emotional events and emotional reactions and second relationship between emotions and individual behaviors (Weiss and Cropanzano, 1996); thus, AET suggests that the subsequent behavior of employees is likely to be a negative response to negative affect. From this perspective, Amabile et al. (2005) believed that emotion-related environmental events would affect individual creative behaviors. Negative affect can trigger avoidance behaviors in order to protect individuals from risky behavior, which would affect creativity, as creativity is such a “trial-and-error” risky behavior (Shalley and Gilson, 2004). Additionally, negative affect could inhibit individual creativity because it will reduce the individual’s cognitive abilities and scope of ideas (Fredrickson, 2001). Negative affect is seen as a decisive factor in employee creative behaviors and this conclusion has also been verified in the literature.

Previous research on abusive supervision has utilized AET to explain the connection between abusive supervision and deviant behavior in the workplace (Mitchell and Ambrose, 2007). Judge et al. (2006) found that hostility, a type of negative affect, had a significant effect on interpersonal fairness, job satisfaction, and abnormal behavior in the workplace. Based on AET, Zhang et al. (2015) found that negative emotions/affect completely mediated the relationship between abusive supervision and user resistance. Sun et al. (2014) demonstrated that negative emotions/affect played a mediating role between abusive supervision and deviant behavior. A conclusion to be drawn from the above evidence is that the effects of abusive supervision on employee’s behaviors are mediated by negative emotions/affect.

Based on the above discussion, AET can explain the mechanism of how abusive supervision is harmful to employees’ creativity. Therefore, we present the following hypothesis: negative affect mediates the relationship between abusive supervision and employee creativity (*hypothesis 1*).

### Moderating Role of Interpersonal Harmony

Harmony is rooted in Chinese traditional culture. The most prominent features of Chinese traditional culture are collective-centered and play a dominant role in an individual’s life, creating an in-group collectivism unique to Chinese society (Farh et al., 1997, 2004). Maintaining interpersonal harmony through in-group collectivism can help to coordinate conflicts, create an environment of trust, improve employee satisfaction with the team, and improve organizational commitment. Scholars have concluded that harmony presupposes the value of differences and advocates the maintenance of harmony through active reconciliation of differences (Leung et al., 2002, 2011; Leung and Brew, 2009). Chen et al. (2015) proposed the dualistic model of harmony enhancement and disintegration avoidance. The former represents a tendency toward building truly harmonious, respectful relationships that guide people to deal with differences and conflicts productively, while the latter represents an avoidance tendency toward conflict and interpersonal disintegration. According to the above analysis, harmony generally has two characteristics: acknowledgment of difference (the premise) and self-regulation (the ability to adapt to organizational contexts). Harmony emphasizes individual self-regulation, i.e., correcting self-behavior through feedback mechanisms such as self-reflection and observation of the situation.

Interpersonal harmony is a precious cultural tradition in China, which promotes and preserves harmonious relations. Farh et al. (1997) thought of interpersonal harmony as a discretionary behavior of an employee, a behavior that avoids the pursuit of personal power and interests, which may adversely affect others or the organization. To be more specific, interpersonal harmony refers to a tendency where people with different personalities seek agreement while shelving differences, thereby resolving conflicts and achieving an overall balance in the fundamental interests of interpersonal communication.

According to cognitive evaluation theory of the emotions, the generation of emotion, except for physiological reaction, goes through several processes: “stimulus situation–evaluation–(interests)–emotion (positive or negative)–behavior (active or passive).” The influence of the independent variable “stimulus event” on the outcome variable of “emotional and behavioral response mode” depends on the individual’s cognitive evaluation mode and the strength of the individual’s coping ability. As a significant negative stressor, abusive supervision creates bad emotional experiences for employees and negative behavior (Tepper, 2007). Therefore, the influence of abusive supervision as a stimulus situation on an individual’s emotions is closely related to the individual’s emotional characteristics and the nature of the cognitive evaluation of the negative stimulus. Based on the emotion regulation process model (Gross and John, 2003), interpersonal harmony as a kind of positive cognitive appraisal method is very important as an “understanding reappraisal” strategy, which can reduce and defuse negative affect and avoid negative behavior. In other words, employees with high interpersonal harmony will react differently to the same abusive supervision compared to those with low interpersonal harmony. When subjected to abusive supervision, individuals with high interpersonal harmony will not allow their actions to be dominated by negative affect, but will take a forgiving attitude after re-evaluating the consequences of their actions; hence, they are less likely to have negative affect. The reason may be that people of high interpersonal harmony are likely to attribute the abusive behavior to the position occupied by the abuser, whereby the supervisor is accorded the right to use their position of dominance because of the position they occupy. In contrast, individuals with low interpersonal harmony may tend to focus on the abusive behavior itself due to its attribution to the abuser; hence, they are more likely to have negative affect in the face of abusive supervision. We conclude, therefore, that harmony is likely to have an impact on employees’ perceptions and will regulate their behavior and, thus, will moderate their reactions to abusive behavior. Hence, we propose the following hypothesis: interpersonal harmony negatively moderates the impact of abusive supervision on negative affect, i.e., abusive supervision will have a lower positive impact on negative affect for employees with high interpersonal harmony (*hypothesis 2*).

**FIGURE 1:**
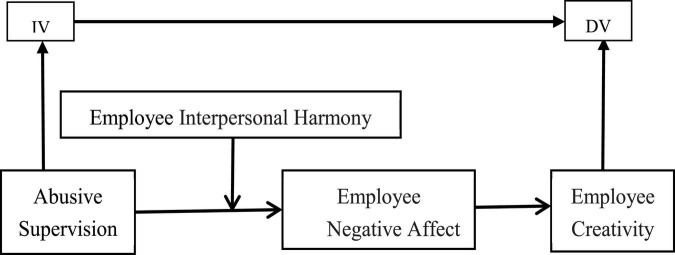
The conceptual model.

Following this overview and analysis of the core variables, a theoretical model can be constructed from the hypotheses. The model is shown in Figure 1.

## Materials and Methods

### Sample and Procedures

The full set of participants consisted of 650 employees who had graduated from colleges or universities in the previous 10 years and represented a sample of the “new generation” of employees. This generation, born in the 1980–1990s, is generally highly educated, open-minded, expected to be respected at work, ready to hop to other jobs, impatient of heavy work demands from supervisors (Pun and Lu, 2010), and form over 60% of the Chinese workforce. Therefore, the degree of their creativity directly affects an enterprise’s creative ability and, hence, the interest in this investigation in verifying how this “new generation” copes with abusive supervision in the Chinese workplace.

Three waves of data collection were applied in this study to reduce the potential common method variance (CMV) (Podsakoff et al., 2007). Data were collected from 398 employees and their direct supervisors. Coded questionnaires on the topics of abusive supervision, negative affect, and interpersonal harmony were filled out by the employees themselves. Creativity questionnaires in line with the employees’ questionnaire codes were filled out by the employees’ direct supervisors. Paired data of supervisor–employee data was, thus, formed to avoid homologous bias. In the first survey, the employees reported their demographic characteristics and perceived abusive supervision. In the second survey, the employees provided their negative affect, interpersonal harmony. In the third survey, direct supervisors were surveyed to evaluate the creativity of their subordinates after their employees had completed the two questionnaires in their entirety. The first two waves of questionnaires were coded one by one before they were distributed, so as to match the answers of their supervisors. Data were collected through the following procedures. In the first week, questionnaires were sent to participating employees via WeChat, a social media platform that provides instant messaging services. In the following 2 weeks, the second questionnaire was sent to the employees who had finished the first survey. Lastly, human resource managers helped us to organize their immediate supervisors and match their questionnaires with their subordinates (one supervisor was matched with only 2–5 subordinates).

The participants in this study were tech employees at internet companies in the Zhejiang province, where Zhejiang has featured commerce and trade for 50 years; further, people in Zhejiang keep pace with the times and have maintained innovative practices through the generations. All (employees and supervisors) were engaged in computer software (system analysis, computer programming), computer hardware (computer maintenance), network (network engineering, network system design), information systems (database management system), and manufacturing (semiconductor device testing). These employees were encouraged to develop creative methods to improve the production process, which required them to come up with novel and useful ideas.

In the first survey, 683 completed questionnaires were acquired from participating employees (97.6% response rate). In the second survey, 612 completed questionnaires were collected from the employees. The third questionnaire was distributed to 147 direct supervisors of the subordinates who had completed the first two questionnaires. As a result, a final sample was obtained of 398 pairs of supervisor–subordinate dyads (65.0% response rate). Of the 398 employees, 63.0% were female. The average age of the employees was 26.3 years (*SD* = 0.51), 68.2% were aged between 21 and 30 years, 94.4% had an undergraduate degree, and 88.7% of the organizational tenure was 3–5 years.

All the participants were Chinese. The measurements used in this study were originally developed in English. In order to guarantee the equivalent meaning, we abided by the widely used back-translation routine to translate these English measurement items into Chinese (Brislin, 1980). Specifically, measurements were first translated into Chinese and then translated back into English with the assistance of a bilingual management professor. One bilingual management scholar was finally invited to check the English and Chinese versions and made some modifications to avoid discrepancies. To minimize CMV, all the items related to independent variables (abusive supervision, negative affect, and interpersonal harmony) were answered by the employees and the dependent variable (employee creativity) was measured by their immediate supervisor’s response.

### Measures

#### Abusive Supervision

A 15-item scale from Tepper (2000) was adopted to measure abusive supervision, which has been applied extensively (Hoobler and Brass, 2006; Zhang et al., 2015). Abusive supervision in previous studies was scored at the individual level. Items were prefaced with the statement “My supervisor…” Sample items included “Ridicules me,” “Puts me down in front of others,” and “Expresses anger at me when he/she is mad for another reason.” All the items in the questionnaire used a five-point Likert-type scale ranging from 1 (“not at all applicable”) to 5 (“highly applicable”). The Cronbach’s alpha for the scale was 0.96.

#### Employee Creativity

Employee creativity was measured using the 13-item scale first designed by Zhou and George (2001) and applied to Chinese samples by Zhang and Bartol (2010). The measure was rated by their immediate supervisors using 5-point scales. Sample items included “This employee suggests new ways to achieve goals or objectives” and “This employee comes up with new and practical ideas to improve performance.” The scale ranged from 1 (“not at all applicable”) to 5 (“highly applicable”). The Cronbach’s alpha of the total 13 items was 0.92.

#### Employee Negative Affect

Negative affect was measured by the Positive Affect and Negative Affect Scale (PANAS) developed by Watson et al. (1988) and containing 10 items. Sample items were “distressed,” “scared,” “hostile,” and “nervous.” The Cronbach’s alpha of the scale was 0.948 and the average variance extracted (AVE) was 0.75, showing good reliability and validity.

#### Employee Interpersonal Harmony

Interpersonal harmony was measured using the four-item scale developed by Farh et al. (1997), which has been tested in Beijing, Shanghai, Hangzhou, and Shenzhen (Farh et al., 2004). A 5-point Likert scale was used, ranging from 1 (“not at all applicable”) to 5 (“highly applicable”). The Cronbach’s alpha of the total 13 items was 0.86.

#### Control Variables

Employee demographics such as gender, age, and educational level were seen as controls in our model because these variables can confound the relationship between abusive supervision and employee creativity. A meta-analysis has suggested that employees who are male, younger, or with a longer tenure are more likely to be targets of workplace bullying (Bowling and Beehr, 2006). Similarly, research has demonstrated that younger or male employees suffer from abuse more frequently than older or female colleagues (Bamberger and Bacharach, 2006). Gender, age, and the education of employees tend to be linked to their creativity (Zhang and Bartol, 2010).

## Results

Here, we discuss the processing and analysis of the primary data gathered from the surveys. First, Statistical Package for the Social Sciences (SPSS) version 21.0 software was used to test the reliability of the scales. Second, SPSS was used to analyze the validity of the scales. The data homology deviation test was then carried out and the proposed hypotheses were tested. The results of the tests are discussed below.

The Cronbach’s alpha values of the scales were greater than 0.70, indicating that all the scales had good internal consistency. The details are as follows: abusive supervision = 0.92, negative affect = 0.95, interpersonal harmony = 0.92, and creativity = 0.86. Confirmatory factor analysis (CFA) was performed on the employee survey data (Table 1) as the preliminary analysis in this study. The results are shown in Table 2. Compared with other models, the data fitting degree of the four-factor model was the best (X^2^/df = 2.21, comparative fit index (CFI) = 0.89, Tucker–Lewis Index (TLI) = 0.91, normed fit index (NFI) = 0.90, and root mean square error of approximation (RMSEA) = 0.0). The results show that the variables have an acceptable discriminant validity and control the homology deviation to some extent.

**TABLE 1:** Discriminant validity analysis of variables.

Models	X^2^/df	CFI	TLI	NFI	RMSEA
4-factor Model	2.21	0.89	0.91	0.9	0.04
3-factor Model	7.03	0.73	0.78	0.88	0.13
2-factor Model	4.4	0.23	0.63	0.59	0.13
1-factor Model	2.05	0.22	0.32	0.24	0.11

*4-factor model 2: abusive supervision, employee negative affect, employee interpersonal harmony, and employee creativity. 3-factor model 2: abusive supervision, employee negative affect and employee interpersonal harmony, and employee creativity. 2-factor model 2: abusive supervision, employee negative affect and employee interpersonal harmony, and employee creativity. 1-factor model 2: abusive supervision and employee negative affect and employee interpersonal harmony and employee creativity.*

**TABLE 2:** Mean, SD, and correlations among the variables.

Variable	*M*	*SD*	1	2	3	4	5	6	7	8
(1) Age	*26.3*	*0.51*	–							
(2) Gender	*2.57*	*0.78*	–0.06	–						
(3) Education	*2.07*	*0.07*	0.05	–0.38**	–					
(4) Organizational tenure	*2.17*	*1.06*	–0.15**	–0.58**	–0.67**	–				
(5) Abusive supervision	*2.15*	*0.76*	–0.21**	–0.03	–0.03*	–0.03	–			
(6) Employee negative affect	*3.09*	*0.68*	0.11	0.02	0.30**	–0.36**	0.30**	–		
(7) Employee interpersonal harmony	*3.29*	*0.69*	0.07	0.08	0.10**	–0.06	0.52**	–0.09*	–	
(8) Employee creativity	*3.34*	*0.74*	–0.09	–0.04	−0.17**	0.15**	−0.17**	–0.19**	0.31**	–

N = 398. *p < 0.05, **p < 0.01, ***p < 0.001.

We then performed *t*-tests to evaluate the mean differences among variables across time as follows: (1) first, the mean differences of gender, age, education, organizational tenure, abusive supervision, and interpersonal harmony were assessed between the first and second samples; (2) second, the mean differences of gender, age, education, organizational tenure, abusive supervision, interpersonal harmony, and negative affect were assessed between the second and third samples; and (3) the mean differences of gender, age, education background, organizational tenure, abusive supervision, and negative affect were assessed between the second and third samples. All the results of the *t*-tests showed no significant mean differences. The conclusion was drawn that the attrition of participants did not substantially influence the results.

Descriptive statistics are shown in Table 2. Abusive supervision was positively correlated with negative affect (*r* = 0.30, *p* < 0.01), which indicates that the higher the perceived level of abusive supervision, the more likely it is to increase employees’ negative affect. Negative affect was negatively correlated with creativity (*r* = −0.19, *p* < 0.01), which indicates that the higher the negative affect, the more likely the employees are to reduce their innovative behaviors. These results are consistent with and provide initial support for the hypotheses.

The stepwise regression analysis was performed to predict the effects of abusive supervision and interpersonal harmony on negative affect and employee creativity. Complete regression results can be found in Table 3. Hierarchical multiple regression analysis was performed to test the main effect and the mediating effect. Following Baron and Kenny (1986), the analysis was divided into four steps to test the hypotheses. In the first step, abusive supervision (explanatory variable) was introduced into the regression equation to test its influence on employee creativity (explained variable). In the second step, abusive supervision was added into the regression equation after the control variables were added to test its effect on employee negative affect (mediating variable). In the third step, negative affect was introduced into the regression equation to test their effect on employee creativity. In the fourth step, after adding the control variables and explanatory variables, negative affect was introduced into the regression equation to analyze the influence of abusive supervision and negative affect on employee creativity.

**TABLE 3:** Results of hypothesis testing.

Variable	Employee negative affect				Employee creativity			
	M1	M2	M3	M4	M5	M6	M7	M8
Age	0.04	0.03	0.04	0.03	–0.06	–0.06	0.05	0.05
Gender	–0.07	–0.08	–0.07	–0.05	–0.00	–0.07	0.04	0.06
Education	0.01	0.07	0.05	0.06	0.08	0.08	0.08	0.08
Organizational tenure	–0.01	0.05	0.06	0.09	–0.05	–0.04	–0.08	–0.08
Abusive supervision		0.34***	0.36**	0.28**		–0.32***		0.10
Employee negative affect							–0.56***	–0.50***
Employee interpersonal harmony			–0.05**	0.03**				
Abusive supervision * Employee interpersonal harmony				–0.17**				
*R* ^2^	0.03	0.23	0.29	0.25	0.02	0.07	0.13	0.05
Δ*R*^2^	0.12	0.03**	0.08**	0.25*	0.03	0.15**	0.03**	0.05*
*F*	0.65	21.80**	17.09**	16.51**	2.32	5.62**	8.82**	8.42**

*N = 398.*

*p < 0.05, **p < 0.01, ***p < 0.001.

As shown in Table 3, abusive supervision had a negative impact on creativity (*B* = −0.32, *p* < 0.001, model 6). Abusive supervision was positively related to negative affect (*B* = 0.34, *p* < 0.001, model 2). Moreover, negative affect was negatively related to employee creativity (*B* = −0.56, *p* < 0.001, model 7). When negative affect was added in, the relationship between abusive supervision and creativity became non-significant (*B* = 0.10, model 8), which shows that negative affect plays a full mediating role between abusive supervision and employee creativity. Nevertheless, negative affect still remained negatively related to creativity (*B* = −0.50, *p* < 0.001, model 8). Thus, hypothesis 1 was supported.

Hypothesis 2 predicts that interpersonal harmony moderates the mediation link of abusive supervision–negative affect–creativity. The general path analytic framework of Edwards and Lambert (2007) was employed to test our hypothesis. The results (Table 4) verify that the size of the difference in the indirect effect of abusive supervision on creativity was 0.09, with the 99% CIs calculated using bootstrap estimates excluding zero. Specifically, the indirect effect of negative affect on the relationship between abusive supervision and employee creativity was significantly weaker at a high level of interpersonal harmony. As shown in Table 3, the interaction between abusive supervision and interpersonal harmony was negatively related to negative affect (*B* = −0.17, *p* < 0.001, model 4).

**TABLE 4:** The moderated mediation effects of abusive supervision.

Employee interpersonal harmony (Mo)	Abusive supervision (X)– employee negative affect (Me)–employee creativity (Y)			
	Stage		Effect	
	1st P_MX_ | 2nd P_YM_		Direct Effects P_YX_	Indirect Effects P_MX_* P_YM_
Simple paths for Low Mo (-1 SD)	0.25**	–0.37**	–0.13	–0.09**
Simple paths for High Mo (+1 SD)	0.17**	–0.15**	–0.04	–0.03
Differences	–0.08**	–0.22	0.09	0.06**

*N = 398.*

**p ≤ 0.01, *p ≤ 0.05 (two-tailed).

*P_MX_, path from abusive supervision to negative affect; P_YM_, path from negative affect to creativity; P_YX_, path from abusive supervision to creativity.*

*Low interpersonal harmony refers to one SD below the mean of interpersonal harmony. High interpersonal harmony refers to one SD above the mean of core interpersonal harmony. Tests of differences for the indirect effects are based on bias-corrected CIs derived from bootstrap estimates.*

To represent the moderating effect of interpersonal harmony on the relationship between abusive supervision and negative affect, we draw an interaction diagram of abusive supervision and interpersonal harmony according to the mean value of interpersonal harmony and the group with one SD above the mean (high interpersonal harmony) and the group with one SD below the mean (low interpersonal harmony).

**FIGURE 2:**
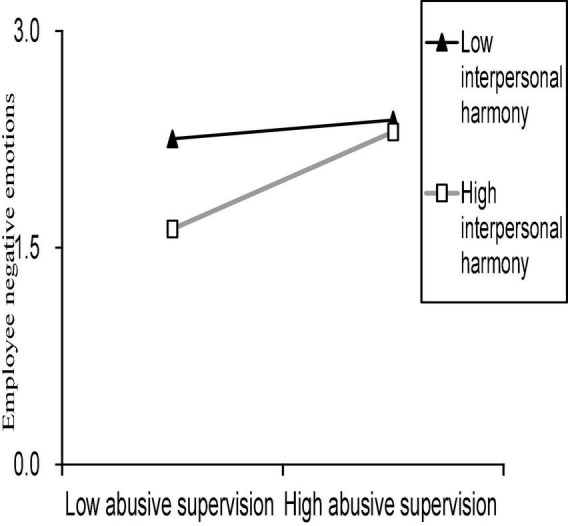
The moderating role of interpersonal harmony.

The interaction effects were plotted according to Stone and Hollenbeck’s (1989) procedure. As can be seen from Figure 2, interpersonal harmony did not change the direction of abusive supervision and negative affect. That is to say, no matter whether the score of interpersonal harmony was high or low, negative affect increased with an increase in abusive supervision. At all the levels of abusive supervision, however, employees with higher interpersonal harmony will have lower negative affect than those with lower interpersonal harmony, which supports hypothesis 2.

## Discussion

This study has empirically analyzed the significant negative correlations of abusive supervision and employee creativity. It is likely that the abusive behavior of their supervisors will lead to the frustration of the subordinates’ enthusiasm for creative behavior, which will lead to a failure in the transformation of innovative ideas into innovative results. The results of this study show that abusive supervision has a significant negative impact on negative affect, indicating that leaders’ abusive behavior will lead to a decline in employees’ emotional states, leading to an increase in employees’ psychological pressure. The findings show that negative affective states failed to make a significant positive contribution to creativity. This finding is consistent with a previous study (Kaufmann and Vosburg, 2002). Results show that negative affect forms the mechanism that can explain the influence of abusive supervision on employee creativity; employees’ affect are affected by organizational factors and then affect employees’ behaviors.

The results also show that interpersonal harmony plays a moderating role on the impact of abusive supervision. Interpersonal harmony attenuates the negative affect of abusive supervision on creative behavior via negative affect. For employees with high levels of interpersonal harmony, the moderation effect is significant. For those with low levels, the effect is non-significant. Interpersonal harmony represents an optimistic and open attitude and plays an important role in dealing with interpersonal conflict in the workplace.

Creativity performs an extremely important role in the sustainable development of organizations (Shen et al., 2020) and how to stimulate employee creativity is becoming increasingly urgent for their employers. Previous research on employee creativity has focused mainly on internal psychological factors such as individual cognition, attitudes, and motivation. This study has built a model to examine the interaction of abusive supervision, negative affect, interpersonal harmony, and employee creativity. We tested our models by using data gathered by a field study. The results show that the abusive behaviors of supervisors will significantly negatively affect the innovation behaviors of employees, from which we can conclude that abusive supervision is one of the important factors affecting employee creativity.

Negative affect mediates the relationship between abusive supervision and creativity with pronounced negative impacts for employees with low interpersonal harmony. By focusing on mediating and moderating effects, our model helps to explain how abusive supervision counteracts creativity and how interpersonal harmony copes with abusive supervision and ensures employee creativity, with negative affect having a mediating effect. Abusive supervision and leadership style influence the emotional states of employees and then reduce their creativity. Therefore, organizations should pay particular attention to the cognition of employees’ emotions and leaders at all the levels should consider taking measures such as caring for employees’ vital interests to reduce their negative affect and, thereby, improve their creativity.

In addition, this study found that interpersonal harmony had a moderating effect. Under the condition of low interpersonal harmony, the negative effect of abusive leadership on employees’ affect is more intense, so organizations should understand the status of their employees’ interpersonal harmony, paying attention especially to the choice of management staff with low interpersonal harmony, thereby creating a positive and harmonious environment of enterprise, improving their employees’ awareness of their affect, and enhancing their creative capabilities for innovation and development.

### Theoretical and Managerial Implications

This study makes several theoretical contributions to the literature. First, we broaden the literature by building an emotional model of how abusive supervision may weaken employee creativity. The implications of abusive supervision on employees’ behaviors in the workplace have been extensively studied (Mackey et al., 2020), but we extend the literature on the impact on creativity by identifying negative affect as an underlying mechanism. This emotional model integrates abusive supervision with employee creativity, within which abusive supervision serves to undermine employees’ affective states, particularly for those who are sensitive to negative external evaluation. Aryee et al. (2007) have demonstrated the link between abusive supervision and the impact on employees’ emotional resources, but it is still unclear how employees who are exposed to abusive supervision suffer a reduction in their creativity. This emotional model of abusive supervision provides new evidence for a mediator associating unfriendly social-environmental situations with employees’ behaviors in the workplace. The implication is that managers should try to eliminate employees’ negative feelings through affective support and interpersonal harmony, thereby encourage creative behaviors.

Second, AET was adopted to help us to better understand the underlying mechanisms and boundary conditions whereby employee creativity is undermined by abusive supervision. Previous studies have provided a social exchange model of creativity (Shen et al., 2020) and a correlation index (COR) theory model (Akram et al., 2021) to explain why abused employees may be less creative at work. Our AET perspective shows that abusive supervision triggers negative affect, which undermines employee creativity. AET, as a relatively new theory, plays a unique role in explaining the relationship between the organizational environment (the workplace) and its members’ attitudes and behaviors. AET reveals that the emotions/affect of employees are affected by work events, especially negative work events, which may lead to a series of changes in emotional states and behaviors. Hence, managers should maintain a high sense of alertness, adjust their leadership style accordingly, and guide employees in managing their emotions and dealing with negative work events in an appropriate way, so as to promote an overall improvement in organizational performance.

Third, the examination of the moderating role of interpersonal harmony reveals the complexity of the relationships among abusive supervision, negative affect, and employee creativity. The findings extend previous studies on the moderating role of cultural values and contextual features. Interpersonal harmony is an interesting moderator because individuals with low interpersonal harmony are vulnerable to external stimuli and the effects of abusive supervision on negative affect are enhanced. The main effect of abusive supervision, which represents a negative evaluation of employees, undermines employees’ affect and creativity at all the levels of interpersonal harmony, but its impact is most destructive for those with low interpersonal harmony. The person–context approach of Kacmar et al. (2009) indicated that the interaction of individual–contextual characteristics can improve the predictive ability of behavioral models for the workplace. It is not surprising, therefore, that our findings show that the mediating role of negative affect is moderated by interpersonal harmony. Thus, cultural values should be viewed as a key moderator that offers boundary conditions for the mediating role of negative affect in the link between workplace atmosphere and creativity. The buffering effect of interpersonal harmony is, therefore, of great significance for research on the intersection of abusive supervision and creativity in the workplace.

### Limitations and Future Directions

As with other studies, this study has some limitations. First, the participants in this study were limited to one targeted sample in a specific region. Although it is representative from the perspective of regional distribution, it is still limited across the whole of China. Moreover, female employees account for the majority of the sample in this study and gender difference may lead to differentiated perception of abusive supervision, which, in turn, may affect the accuracy of the results. Although gender was used as a control variable in order to reduce its impact on the results, the role of gender in abusive supervision and employee effectiveness still needs further consideration in future studies (Whitman et al., 2014). Additionally, the sample size was not large enough to meet our target. The response rate was only 65.0% for the supervisor–subordinate dyads in the third wave.

Second, this study explores the effect of abusive supervision on employee creativity at the individual level, but team abusive supervision and team creativity were not involved in this study. The relationship between team abusive supervision and team creativity is worth considering in future research. Additionally, this study focuses only on the relationship between abusive supervision and employee creativity as well as the mediating and moderating mechanisms. Future studies could explore the impact mechanisms of abusive supervision on individual organizational citizenship behavior, team organizational citizenship behavior, and other behaviors outside of the workplace.

Third, the sample in this study was taken from Chinese enterprises with high power distance and traditional culture. Such a cultural background may affect employees’ attitudes and behavioral responses to abusive supervision. Therefore, future studies need to take cultural values such as power distance and collectivism vs. individualism as regulating variables, both at the individual level and at the team level, to further test the consistency and applicability of this study’s findings to different cultural situations.

## Data Availability Statement

The raw data supporting the conclusions of this article will be made available by the authors, without undue reservation.

## Ethics Statement

The studies involving human participants were reviewed and approved by Zhejiang Shuren University Research Ethics Review Committee. The patients/participants provided their written informed consent to participate in this study.

## Author Contributions

CH: conceptualization and original draft preparation. ZY and ZS: methodology, analysis, validation, review, and editing. HZ: supervision. All authors contributed to the article and approved the submitted version.

## Conflict of Interest

The authors declare that the research was conducted in the absence of any commercial or financial relationships that could be construed as a potential conflict of interest.

## Publisher’s Note

All claims expressed in this article are solely those of the authors and do not necessarily represent those of their affiliated organizations, or those of the publisher, the editors and the reviewers. Any product that may be evaluated in this article, or claim that may be made by its manufacturer, is not guaranteed or endorsed by the publisher.
